# ^210^Po and ^210^Pb content in the smoke of Heated Tobacco Products versus Conventional Cigarette smoking

**DOI:** 10.1038/s41598-022-14200-2

**Published:** 2022-06-20

**Authors:** Aurélie Berthet, Audrey Butty, Jérémie Rossier, Isabelle Jacot Sadowski, Pascal Froidevaux

**Affiliations:** 1 University of Lausanne, Ctr Primary Care & Publ Hlth Unisante, 1010 Lausanne, Switzerland; 2 Institute of Radiation Physics, Lausanne University Hospital and University of Lausanne, Grand Pré 1, 1007 Lausanne, Switzerland

**Keywords:** Biomarkers, Risk factors, Analytical chemistry

## Abstract

^210^Po is a radioactive component of conventional cigarette tobacco smoke and is a recognized carcinogen. Despite the expanding market of heated tobacco products, no data are available on the activity of ^210^Po in the smoke of IQOS Heets cigarette. We determined the ^210^Po activity in the mainstream smoke of thirteen cigarette brands available on the Swiss market using a smoking machine and compared the results to the ^210^Po activity measured in the mainstream smoke of the IQOS system. In addition, we measured the ^210^Po and ^210^Pb loss on heating after uniform heating from 50 to 600 °C for several cigarette brands and the Heets cigarettes. 13.6 ± 4.1% of ^210^Po activity was found in the mainstream smoke in conventional cigarette smoking (7% for ^210^Pb). This dropped to 1.8 ± 0.3% in the mainstream smoke of IQOS Heets. Conversely, when the tobacco was heated uniformly at 330 °C, a loss of ^210^Po of more than 80% was observed for all type of cigarettes. Apparently, IQOS significantly reduced the ^210^Po and ^210^Pb activities in the mainstream smoke. However, our results show that only 15% of the Heets tobacco reaches 330 °C with IQOS. While IQOS reduces the ^210^Po and ^210^Pb activities in the mainstream smoke compared to conventional cigarettes, it only heats a marginal fraction of the tobacco present in the Heets cigarette. Because smoking is an addiction (mostly due to nicotine), IQOS could possibly deliver an unsatisfactory dose of nicotine to a Heets cigarette smoker, as most of the tobacco is left unaltered.

## Introduction

Polonium-210 **(**^210^Po) and lead-210 (^210^Pb) are natural radionuclides of the uranium (^238^U) decay series, which are present within and on the surface of tobacco leaves^1–5^. A large part of the ^210^Po and ^210^Pb activity in tobacco originates from the capture of radon-222 (^222^Rn) progeny aerosols by the trichomes of leaves, whilst a smaller part originates from root transfer^1,3,6^. Owing to the significant volatility of Po and Pb in aerosol particles, both radionuclides are present in mainstream cigarette smoke^2,4,5,7–9^. ^210^Po, as an energetic α-particle emitter, has readily been recognized as a potential carcinogenic component of the tobacco smoke^10–12^. As early as 1964, Radford and Hunt hypothesized that the presence of ^210^Po in tobacco smoke, and its preferential localization in the bronchial epithelium, would be a cause of lung cancer^5,13^. Despite studies documenting the appearance of lung cancer in animals exposed to low dose of ^210^Po^12^, the tobacco industry made no successful effort to remove ^210^Po and ^210^Pb from tobacco^7,14,15^. Studies on the role of ^222^Rn and its progeny as a cause of lung cancer have demonstrated a synergetic effect with tobacco smoke, with efforts to reduce in-house ^222^Rn beneficiating more to smokers^16–18^. In this respect, it appears that ^210^Po present in tobacco smoke could be responsible of some lung cancers otherwise attributed to radon^10,15^.

^210^Po in tobacco smoke commits an effective radiation dose to the lung, which could be close to the annual dose limit of 1 mSv for heavy smokers, depending on the dose assessment method used. If the annual effective dose exceeds 1 mSv, most national regulations would require marking the cigarette packs with a radioactive hazard label or corresponding pictograms to inform users of the radioactive risk^8,19^. As a matter of fact, Winters and Difranza reported “having received hundreds of phone calls from smokers who quit on learning about alpha radiation in cigarette smoke”, after the publication of a letter denouncing the lack of research on ^210^Po in tobacco in The New England Journal of Medicine^2,7,20^. Thus, the presence of radioactivity in tobacco products and tobacco smoke could be a valuable argument for smoking cessation and could be used as an additional public heath intervention to reduce smoking prevalence tobacco-related diseases.

Heated tobacco product (HTP) is a newer way of smoking, in which the tobacco is heated at lower temperatures than a conventional cigarette. HTP is an expanding market within the cigarette industry, with significant interest from different demographics including non-smokers. The most broadly available on the market and widely used product is IQOS, developed by Philip Morris International (PMI). IQOS is an electronic system in which the tobacco is heated at 330 °C. At this temperature, the possibility of ^210^Po and ^210^Pb to volatilize through aerosol smoke particles is questioned. To provide insight on this, we designed a study aimed at determining the activity of ^210^Po and ^210^Pb in tobacco and tobacco mainstream smoke for conventional cigarette brands sold in Switzerland, the reference cigarette 1R6F, and the Heets cigarette. In addition, we determined the ^210^Po and ^210^Pb loss on heating at different temperatures (50–600 °C) to define the temperature of half-loss (T_1/2_) for both radionuclides when the cigarette is heated uniformly. Combining these results, we were able to define the quantity of ^210^Po and ^210^Pb present in the mainstream smoke of HTP (IQOS) system and to quantify the percentage of the tobacco mass really heated to the target temperature of 330 °C in the IQOS system. To fill this currently missing knowledge on smoker exposure to ^210^Po and ^210^Pb will inform on the potential radiation dose to the lung in cigarette and Heets smokers.

## Results

### ^210^Po and ^210^Pb activity in conventional cigarettes, 1R6F and Heets

Thirteen cigarette brands, the 1R6F reference cigarette, the IQOS Heet bronze label, some CBD products, and some loose tobacco samples were tested to quantify their ^210^Po content (Table 1). The ^210^Po activity was remarkably constant in tobacco conventional cigarette fillers, with an average activity of 15.0 ± 2.3 mBq per cigarette or 25.2 ± 2.6 mBq g^−1^ tobacco (*n* = 15). Loose tobacco samples have similar results, with an average activity of 22.5 ± 1.5 mBq g^−1^ tobacco (n = 3). Only CBD samples, containing no tobacco per se, had a low ^210^Po activity of 1.8 ± 0.2 mBq g^−1^ product. Al Capone CBD cigarillos contains a mixture of tobacco and CBD, and thus, contained a ^210^Po activity close to the tobacco samples (22.3 ± 1.0 mBq g^−1^ product) because of the low CBD content. IQOS Heets bronze label had an equivalent ^210^Po content to conventional cigarettes, or 23.7 ± 2.1 mBq.g^-1^ tobacco, demonstrating a similar tobacco origin than conventional cigarettes. The analysis of ^210^Pb with the double-spike method showed that the ^210^Po activity is supported by a similar activity of ^210^Pb (^210^Po/^210^Pb ratio of 1.06 ± 0.05). This demonstrates that ^210^Po and ^210^Pb are in secular equilibrium in tobacco available on the Swiss market.

**Table 1:** ^210^Po activity (mBq per cigarette and mBq g^−1^ tobacco, n = 5) in the tobacco fillers of cigarettes and in mixed tobacco and CBD products sold in Switzerland.

Tobacco product	^210^Po unit activity (mBq/cig)	^210^Po mass activity (mBq g^−1^)
**Conventional cigarettes**		
Camel Filter	15.8 ± 2.0	26.0 ± 3.4
L&M Red Label	13.4 ± 2.6	23.4 ± 3.9
Lucky Strike Red	16.6 ± 1.3	24.8 ± 2.0
Kent Taste +	15.2 ± 0.8	26.9 ± 0.9
Philip Morris Quantum Blue	12.7 ± 0.8	24.2 ± 1.0
Marylong Filter	17.1 ± 0.7	28.0 ± 1.7
Winston Blue	13.5 ± 1.0	27.3 ± 2.1
Marlboro Red	12.7 ± 1.6	21.0 ± 3.2
Parisienne Yellow	13.9 ± 1.3	21.9 ± 2.1
Chesterfield Original	14.1 ± 0.6	27.1 ± 0.9
Benson & Hedge (White/Blue)	13.6 ± 2.7	24.3 ± 4.1
Natural American Spirit Yellow	15.8 ± 3.6	24.9 ± 5.8
Gauloises Bleues	21.2 ± 2.0	30.1 ± 2.8
Research Cigarettes IR6F	14.0 ± 0.2	22.3 ± 0.4
**Heated tobacco product (HTP)**		
IQOS Heets bronze label^a^	6.9 ± 0.4	23.7 ± 2.1
**Tobacco and CBD products**		
Fleur du Pays N°1 (Yellow)	–	24.5 ± 0.8
DRUM the Original		20.8 ± 3.2
C Pure Fedtonic Fleurs (CBD)		1.8 ± 0.2
Al Capone Cigarillos (CBD)^b)^	–	22.3 ± 1.0
Cigar Villiger No 7^c)^	–	15.0 ± 0.4

The given values are an average of five replicates per tobacco products, each replicate came from the same pack bought in a local tobacco shop (Lausanne, Switzerland).

^a^IQOS Heets cigarette contains on average 290 mg of tobacco compared to 600 mg for conventional cigarettes.

^b^A cigarillos Al Capone CBD (a mixture of tobacco and CBD) weighs on average 1240 mg.

^c^A cigar Villiger N°7 weighs on average 4330 mg.

### ^210^Po and ^210^Pb in mainstream smoke

^210^Po and ^210^Pb were washed from the smoke using the three sequential 1 M HCl washing flasks F1 to F3. Results from independent analysis of each flask fraction showed that F1 retained about 70%, F2 about 20% and F3 about 10% of total ^210^Po activity in the mainstream smoke from conventional cigarettes (Table 2). In this respect, three washing flasks seem satisfactory to retain more than 95% of the ^210^Po present in the mainstream smoke. The ^210^Po content in the ashes represented about 20% of the total ^210^Po activity, and it is similar to the filter. The ^210^Po budget showed that side-stream ^210^Po represented almost half the ^210^Po activity of a conventional cigarette, demonstrating that passive smoke contains the radiotoxic ^210^Po. The results of the 24 experiments using nine different cigarette brands, including 1R6F, showed that 13.6 ± 4.2% (*n* = 24) of total ^210^Po present in the cigarette was transferred to the mainstream. Results were very different for IQOS. The majority of ^210^Po is recovered in the residue of the Heets cigarettes retrieved from the system after smoking (78%). The activity measured in the mainstream smoke represented 1.8 ± 0.3% (n = 8) of the total ^210^Po present in the Heets cigarettes while the activity measured in the side-stream represented about 8% of total ^210^Po. However, the mainstream smoke of IQOS was mostly formed by a glycerol aerosol, which was somewhat different to conventional cigarettes. This glycerol aerosol did not mix well with 1 M HCl washing; therefore, we adapted the protocol to washing solution containing oxidative reagent such as KMnO_4_, the Fenton’s reagent and the Jones’s reactant (H_2_SO_4_/K_2_CrO_3_) as powerful alcohol oxidant. Nevertheless, the results were very similar to 1 M HCl washing solution, showing that all the ^210^Po activity was measured in IQOS mainstream, regardless of the choice of the washing solution.

**Table 2:** Percentage (%) of the ^210^Po activity measured in a given component part of tobacco products, namely ashes, filters, and mainstream smokes, compared to the total activity contained in the tobacco filler of cigarette.

Component part	Camel filter (% of ^210^Po activity)	Marlboro red (% of ^210^Po activity)	Lucky strike (% of ^210^Po activity)	IQOS (% of ^210^Po activity)
Ashes (or residue for IQOS)	19.0 ± 5.0	18.1 ± 4.1	16.5 ± 4.3	78.1 ± 5.3
Filter	19.0 ± 1.0	20.4 ± 6.1	18.2 ± 4.3	10.9 ± 1.3
**Mainstream**^a^				
*F1*	67.0 ± 18.2	64.5 ± 6.8	77.6 ± 15.3	72.7 ± 17.5
*F2*	22.7 ± 8.3	24.1 ± 7.2	12.5 ± 5.2	18.2 ± 7.4
*F3*	10.2 ± 4.2	11.3 ± 4.6	9.8 ± 3.7	9.1 ± 3.9
Total mainstream^b^	13.3 ± 4.1	15.6 ± 4.0	9.4 ± 1.4	1.8 ± 0.3
missing (sidestream)	49.0 ± 8.7	46.0 ± 5.0	55.9 ± 4.0	8.3 ± 4.5

Average value of three experiments using five cigarettes smoked in each experiment. F1: washing flask 1 connected to the conventional cigarettes or to the IQOS Heets bronze label cigarettes; F2: washing flask 2 connected to F1 and F3; F3: washing flask 3 connected to F2 and the smoking machine.

^a)^Percentage of the total mainstream activity as measured in each of the three washing flasks.

^b)^Percentage of the mainstream activity compared to the total activity in the cigarette.

**Figure 1:**
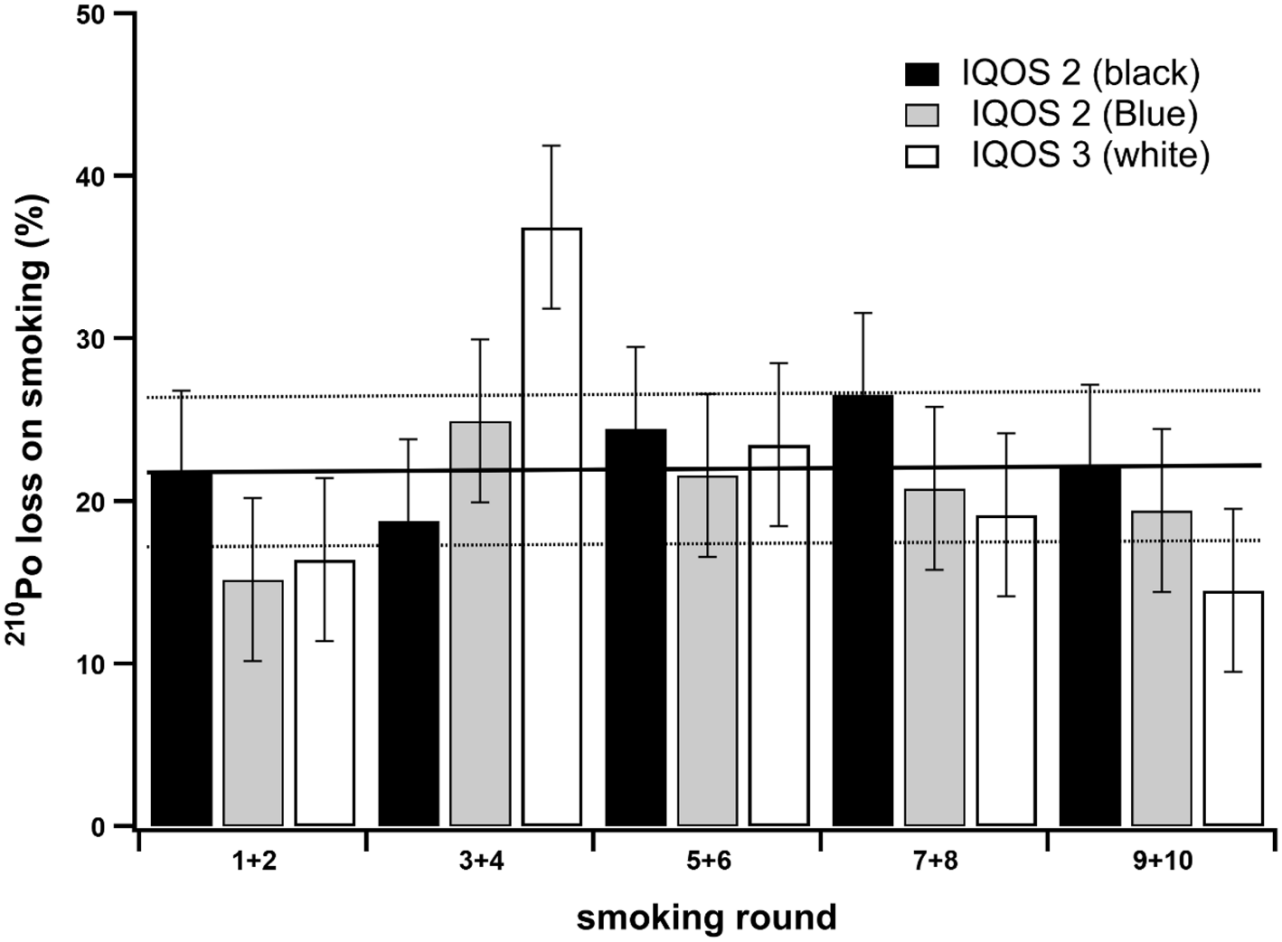
Percentage (%) of ^210^Po lost from the tobacco after smoking a Heets cigarette using two different IQOS generations. 10 cigarettes were smoked and grouped by two before analysis to improve sensitivity. Results from the IQOS 2 are in black and in gray and results from IQOS 3 are in white.

The mainstream smoke of IQOS Heets bronze label was generated using two generations of IQOS systems: IQOS 2 and IQOS 3. The main changes between the two generations are the faster charging time (3:30 min vs 4:10 min for IQOS 3 and IQOS 2, respectively) and the extended battery life for IQOS 3 compared to IQOS 2, the oldest version. The two generations were used to test the reproducibility of the heating temperature by determining the percentage of ^210^Po lost on smoking in ten different smoking runs (two different apparatus for IQOS 2 and one apparatus for IQOS 3). Then, Heets cigarette residues were grouped by two to form one sample, improving sensitivity. Results showed that an average and constant loss of about 21.0 ± 5.0% was observed, regardless the IQOS generation used (Fig. 1).

### ^210^Po and ^210^Pb loss on heating

**Figure 2:**
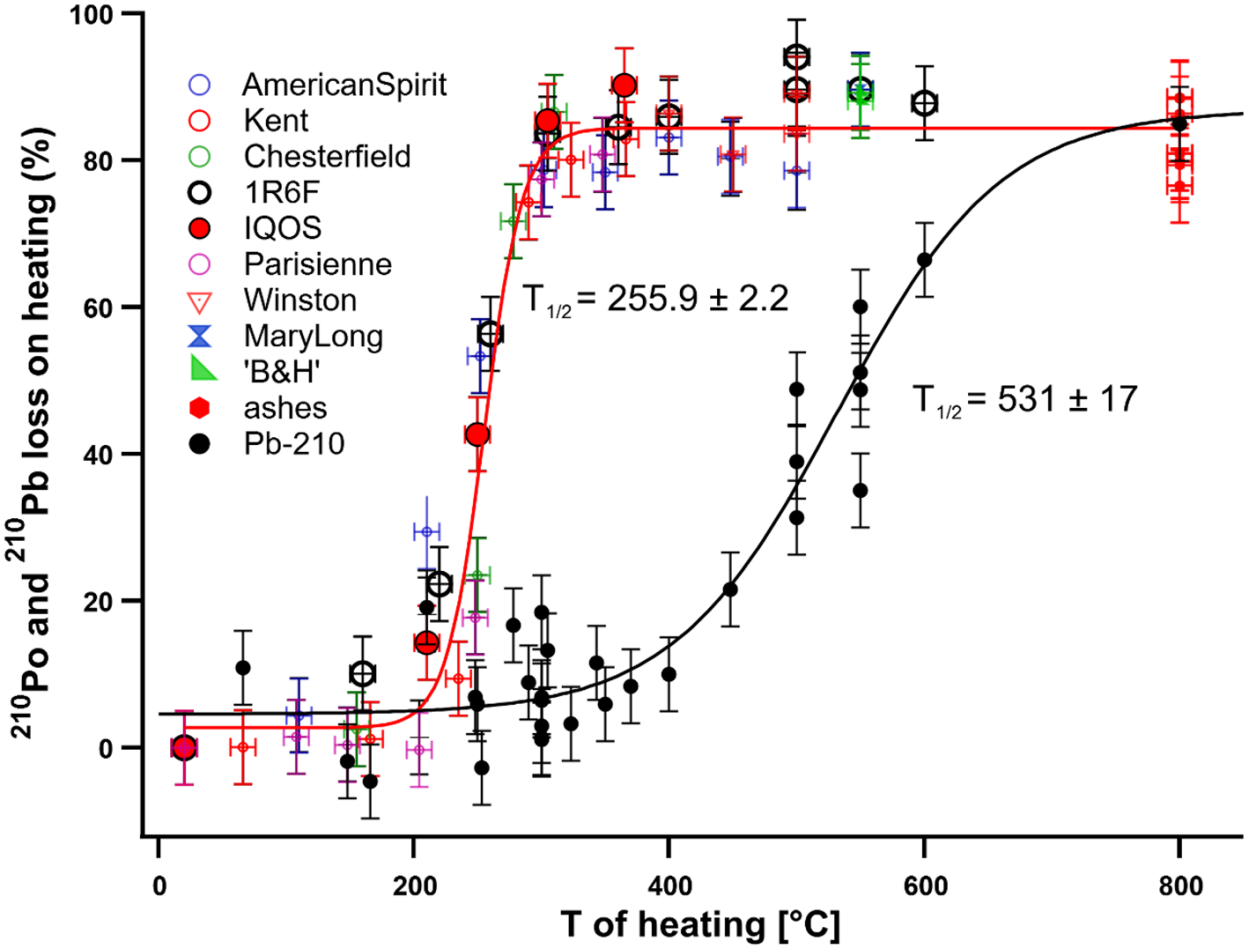
Percentage (%) of ^210^Po and ^210^Pb loss on heating for cigarette heated homogeneously in a copper cylinder. T_1/2_ represents the temperature at which half of the ^210^Po or ^210^Pb activity is lost from the tobacco cigarette filler. 1R6F: large black open circle; Heets: large red closed circle. 800 °C has been fixed as the maximum temperature reached during conventional smoking (ashes retrieved from a conventional smoker).

We used a homemade heating system to determine the temperature of half loss (T_1/2_) of ^210^Po and ^210^Pb from tobacco when the cigarette was heated homogeneously. T_1/2_ represents the temperature at which half of the ^210^Po or ^210^Pb activity is lost from the tobacco cigarette filler. Results are presented in Fig. 2 and showed that 50% of ^210^Po loss already occurred at a temperature of 255.9 ± 2.2 °C, regardless the cigarette brand, including 1R6F reference cigarette, and Heets cigarette. Pb being less volatile than Po, the temperature of half loss of ^210^Pb was significantly higher (531 ± 17 °C). These results showed that when heated homogeneously, the percentage of loss of ^210^Po and ^210^Pb from conventional, reference 1R6F and Heets cigarettes were extremely consistent. Consequently, the lower percentage of ^210^Po present in the mainstream smoke of IQOS Heets was only due to inhomogeneous heating, where a large part of the tobacco was left unaltered after smoking. Based on a 80% loss of ^210^Po at 300 °C for any cigarette (including Heets) and a 2% of ^210^Po activity in the mainstream smoke of IQOS system, we estimated that the IQOS system heated about 15% of the whole tobacco in direct contact with the electrical resistance only.

### ^210^Po supported by ^210^Pb in the mainstream smoke

We measured ^210^Pb (with the double-spike method) in sixteen independent experiments including seven different conventional cigarette brands, the 1R6F reference cigarette and the Heets cigarette. Results showed a large dispersion of values, with minimum value of 38% supported ^210^Po and maximal value of 83% supported ^210^Po for conventional (including 1R6F) cigarettes. On average, 52.1 ± 16.1% (*n* = 16) of supported ^210^Po was in the mainstream smoke. However, when Heets was smoked, we found a higher level of supported ^210^Pb in the mainstream smoke (79.4 ± 35%, *n* = 5). The lower volatility of Pb compared with Po might explain part of the secular disequilibrium in the mainstream smoke compared to tobacco, alongside larger variability in the results. However, results of the loss on heating experiments (Fig. 2) showed that at the temperature of conventional smoking (600–800 °C), the loss of ^210^Pb was above 80%. Nevertheless, the filter could be more efficient to remove ^210^Pb-bearing particles. On the other hand, the higher percentage of supported ^210^Po in the mainstream smoke of the Heets cigarette could be explained by the different composition of the smoke aerosol (glycerol aerosol), which is possibly more efficient to carry ^210^Pb-bearing particles to the mainstream, even at lower temperatures.

## Discussion

Our results show that ^210^Po and ^210^Pb activity in tobacco of conventional cigarettes sold in Switzerland, the 1R6F reference cigarette and Heets cigarette, are very similar for all brands and within the range of ^210^Po activities determined in other similar studies^9,22^. In addition, our results show that ^210^Po and ^210^Pb are in secular radioactivity equilibrium in tobacco fillers. ^210^Pb is less radiotoxic than ^210^Po due to the emission of a low energy α-particle; however, its long physical half-life of 22.3 years (compared to 138.4 days for ^210^Po) makes its presence in lungs a health hazard because it will continually produce the very radiotoxic bismuth-210 (^210^Bi; high energy β-particle > 1 MeV) and ^210^Po radionuclides. Consequently, we may assume that the presence of ^210^Pb in the mainstream smoke and subsequently in the lungs will induce deleterious impact on health.

Conventional cigarettes transfer a fraction of 13.6% of the total ^210^Po cigarette activity in the mainstream smoke. This fraction is within the range of other studies, in which a percentage of transfer between 6 and 20% has been determined^22,23^ and very close to the most recent study^24^ using a smoking machine to determine ^210^Po and ^210^Pb in the mainstream smoke (13% for ^210^Po). We also found that a fraction of 7% of total ^210^Pb is transferred to the mainstream smoke. This percentage is very similar to the 8% value found by Schayer et al.^24^ Thus, our results back up most of the data in the literature on conventional cigarettes and radioactive concentrations of ^210^Pb and ^210^Po. Building on this, our study presents the first data on the IQOS system and the ^210^Po and ^210^Pb radiotoxic components in smoke aerosol. We found that the Heets cigarette contains the same ^210^Po and ^210^Pb activities than the other conventional cigarette brands sold in Switzerland. However, the IQOS system transfers only 2% of the total ^210^Po activity from a Heets cigarette and about 1.5% of its ^210^Pb activity to the mainstream aerosol. In this respect, IQOS seems to be a worthy option to users regarding their exposure to radiotoxic ^210^Po and ^210^Pb. Nevertheless, our study on the homogeneous heating of tobacco establishes also that the percentage loss of ^210^Po and ^210^Pb from tobacco are equivalent, regardless the conventional cigarette brand, 1R6F reference cigarette or Heets cigarette. These results demonstrate that IQOS system heats only a small fraction of the tobacco content at 300 °C, evaluated here at 15%. This fraction represents clearly the tobacco fraction in close contact with the electrical resistance. In this respect, the IQOS system may not deliver a satisfactory pulse of nicotine to users, leading to an over-consumption of Heets cigarettes, with similar health deleterious effect than conventional cigarettes. In our study, the smoking regime used to generate emissions from IQOS was the HCI regime used for conventional cigarettes. However, studies in IQOS users observed a larger mean puff volume (around 60–65 mL) and a shorter puff intervals (about 10–12 s) compared to the HCI standardized regime (puff volume of 55 mL and puff interval of 30 s)^25–27^. This use-behavior difference in IQOS smoker influences the aerosol generated, and might underestimate user exposure to ^210^Po and ^210^Pb^26^. Phillips-Waller et al. compared HnB IQOS, Juul, E-cigarette and conventional smoking in their ability to deliver nicotine and rated the satisfaction of the smoker after overnight abstinence of smoking and vaping^28^. Results show that IQOS delivered less nicotine than conventional smoking and received less favorable ratings than Juul (effect on the urge to smoke), which may be correlated with very partial heating of the tobacco. In addition, Meehan-Atrash et al. found nearly no free-base nicotine in aerosol from HnB IQOS using a sophisticated ^1^H-NRM method^29^. However, a greater fraction of free-base nicotine (α_fb_) is associated with a faster nicotine absorption kinetic and physiological response. The very low concentrations of free-base nicotine in IQOS aerosol confirmed the limited absorption of nicotine compared to conventional cigarettes, and thus the assumption to consume more Heets to obtain a satisfying dose of nicotine.

For members of the public, there is no surprise that tobacco products contain toxic chemicals. However, most individuals are not aware that tobacco products contain radioactive particles, which lodge in the lungs when inhaled. As radioactivity is most feared^30,31^, this knowledge could be an incentive for smoking cessation. The WHO Framework Convention on Tobacco Control recommends information on smoking associated risks, such as information campaigns, as a key measure to reduce tobacco demand^32^. A recent systematic review reports little evidence and no significant effect of biomedical risk assessment on smoking cessation^33^. However, showing to smokers that they had developed atherosclerotic plaques^34^, or motivational intervention with feedback on a combination of, including spirometry, exhaled carbon monoxide and pulmonary symptoms discussion, have increased significantly the smoking cessation rate^35^. In a similar way, we can expect that providing smokers with their personal level of urine ^210^Po radioactivity level, together with a relevant explanation and a motivational intervention, will increase their motivation for smoking cessation. This is particularly important in view of the significant increase in HTP users during the Covid-19 pandemic, as observed by Gallus et al. in a very recent study in Italy^36^. Work is in progress in our laboratory and hospital to study the role of fear of radioactivity in tobacco products (^210^Po) in the smoking cessation rate.

Our results confirmed previous studies showing the presence of ^210^Po and ^210^Pb in the mainstream of tobacco smoke. They also demonstrated that the mainstream smoke of the IQOS system contains ^210^Po and ^210^Pb, although at lower level compared to conventional smoking. This lower percentage of ^210^Po and ^210^Pb in the smoke is related to the small fraction of tobacco really heated at a temperature of 300 °C and not to specific counter-measures, such as tobacco acid-washing, associated with the ^210^Po in tobacco concern. Consequently, tobacco smoke from conventional and Heets cigarettes commit an effective radiation dose to the lung of users that may further result in cancer. Providing that knowledge of radioactivity induced by tobacco products is effective to increase motivation to quit among smokers, this intervention could be cost-effective and have a major public health impact.

## Methods

### Chemicals

Hydrogen peroxide solution (H_2_O_2_ > 35%), ascorbic acid and NH_4_FeSO_4_, KMnO_4_ and K_2_Cr_2_O_7_ salts were purchased from Sigma-Aldrich (Switzerland) and were puriss. p.a. grade. Nitric acid solution (HNO_3_ 65%), Hydrochloric acid solution (35%) and ammonia solution (30%) were purchased from Carlo-Erba (Reactolab, Switzerland) and were analytical grade. ^208^Po and ^209^Po solutions (25 mBq/ml) were obtained from the Metrology group of the Institute of Radiation Physics, Lausanne and were traceable to NIST standard (^209^Po, NIST SRM 4326) or NPL standard (^208^Po, NPL A160443).

### ^210^Po and ^210^Pb in tobacco fillers and loose tobacco

All the tobacco samples used in this study are sold freely and legally in Switzerland. Thirteen brands of conventional cigarettes and IQOS Heets bronze label were purchased from a local tobacco shop in Lausanne, Switzerland (see Table 1 for details). The 1R6F reference cigarettes were purchased from the Kentucky Tobacco Research and Development Center (Lexington, KY, USA). CBD, a cannabidiol product with a THC content less than 1%, is sold legally in Switzerland and was purchased from a specialized shop (Lausanne, Switzerland). The tobacco content of one conventional cigarette (~ 600 mg), one Heets cigarette (~ 290 mg) or 600 mg of loose tobacco sample was weighed in a 100 ml Teflon flask and spiked with 25 mBq of ^209^Po. 20 ml of concentrated HNO_3_ (65%) and 3 ml of 30% H_2_O_2_ were added, and the tobacco was digested in a pressurized microwave apparatus (Milestone UltraClave IV, Germany) under a pressure of 50 bars and at a temperature of 180 °C for 15 min. The solution was poured into a 300 ml beaker and diluted to 250 ml with ultrapure water. Two mg of Fe^3+^ was added and ^210/209^Po and ^210^Pb were co-precipitated on iron hydroxides at pH 8 with addition of 25% NH_4_OH. After decantation and centrifugation, the iron hydroxide precipitate was dissolved in 100 ml of 1 M HCl and 1 g of ascorbic acid was added to reduce Fe^3+^ to Fe^2+^. A silver disk (∅ 1 cm), covered on one face with tape, was suspended in the solution for 2 days, inducing the spontaneous electrochemical deposition of polonium isotopes on the disk. After retrieval, the disk was counted (400,000 to 864,000 s) on a surface barrier detector (PIPS) of 450 mm^2^ in an Alpha Analyst (Canberra-Mirion, France) Alpha Spectrometer. The 1 M HCl fraction, containing ^210^Pb, was kept in a plastic bottle in a refrigerator at 4 °C for at least six months, allowing for ingrowth of ^210^Po from ^210^Pb in the solution. After six months, 1 g of ascorbic acid and 25 mBq of ^208^Po was added (double-spike method) and a new silver disk suspended in the solution for two days before alpha spectrometry counting as described above. ^210^Po activity was calculated based on the ^209^Po tracer activity, while the ^210^Pb activity was determined based on the ^208^Po tracer activity after correction for partial regrowth of ^210^Po calculated using the Bateman equation.

Uncertainties were calculated as the standard deviation of several measurements (most often n = 5) or as the quadratic propagation of uncertainties for individual measure: u(^209^Po): 2%; u(^208^Po): 2%; v(tracer):1%; uncertainty on the number of counts: (N)^1/2^/N.

### ^210^Po and ^210^Pb in the mainstream smoke

We used a smoking device designed in our facility to capture the mainstream aerosol and developed to meet the standards for tobacco cigarettes^21^. The Health Canada Intense (HCI) regime was followed to generate smoke from conventional cigarettes and IQOS system. The cigarette smoke was adsorbed in 1 M HCl in a similar way as described by Kubalek et al.^22^ and Horvàth et al.^37^ Briefly, the cigarette was connected to three 250 ml washing flasks (F1-F3) containing 100 ml of 1 M HCl each and linked in series to trap ^210^Po. The first washing flask (F1) was connected to the cigarette or Heets and the last washing flask (F3) was connected to the smoking machine. Eleven puffs of 55 ml for two seconds duration at a frequency of 30 s were programed for each cigarette, including IQOS. Five replicates of each conventional cigarette brand were smoked and combined into a single sample for increasing sensitivity. Ten replicates were used for the Heets cigarettes. To determine the recovery rate of ^210^Po in the washing flasks, each washing flask was analyzed separately in nine experiments (i.e., three repeated experiments using three different cigarette brands, and five replicates smoked for each cigarette brand per experiment). In the other experiments, the 1 M HCl content of Flasks F1-F3 was combined to make one sample for increasing sensitivity. During the nine independent experiments, the ashes and the filters were gathered and ^210^Po was measured following the described method for tobacco.

^210^Po was measured in the 1 M HCl solution (F1–F3) following the addition of 25 mBq of ^209^Po as radioactive tracer and the destruction of the organic matter (tobacco particulate matter) using the Fenton reaction (addition of 100 mg of NH_4_FeSO_4_ and 20 ml of H_2_O_2_ and heating at 70 °C). After two hours at 70 °C, iron hydroxide precipitation was performed with the addition of 25% NH_4_OH. The precipitate was treated as above to determine the ^210^Po activity and the 1 M HCl fraction was kept for six months for the determination of ^210^Pb with the double-spike method. A similar method was used to determined ^210^Po and ^210^Pb in the mainstream smoke of the IQOS system, except that 10 cigarettes were smoked in a run to make one sample to increase the sensitivity. However, due to the different nature of the IQOS Heets smoke (glycerol aerosol) compared to conventional cigarette smoke, oxidative reagents were also used (KMnO_4_, Fenton’s reagent, Jones’s reagent) to aid the retrieval of ^210^Po from the washing flasks.

### ^210^Po and ^210^Pb percentage loss on heating

We constructed a special device to heat uniformly the tobacco in conventional and Heets cigarettes to determine the loss of ^210^Po and ^210^Pb on heating at various temperature (50–600 °C) as presented in Figure SM1. The heating block is a copper cylinder of the length of a conventional cigarette (excluding the filter), with a hole that tightly encloses a cigarette, and the filter remains outside the cylinder. The copper cylinder was embedded in a heating cord (BriskHeat BWH052020L) connected to a command box (BriskHeat SCDE Temperature Controller) where the required temperature was programmed. The temperature box was controlled by a thermocouple inserted into the center of the tobacco (Figure SM1) which recorded the tobacco temperature during the experiment. The thermocouple controls the temperature box to verify that the targeted temperature was reached and not exceeded. A cigarette from the brand to be tested was used as control to fix the targeted temperature; this cigarette was afterwards removed and replaced by the cigarette to be tested. The cigarette was heated for exactly five minutes; it was then removed from the copper block and sprinkled with water to stop the heating. Heated tobacco was retrieved form the heated cigarette and treated as described above to determine ^210^Po and, for some brands, ^210^Pb. Starting from 620 °C, it was not possible to retrieve the heated cigarette because it started to burn spontaneously. Thus, the curve representing the ^210^Po and ^210^Pb percentage loss on heating stops after 600 °C.

## Supplementary Information

Supplementary Information.

## Data Availability

The datasets generated and analyzed during the current study are not publicly available due to sensitive matter but are available from the corresponding author on reasonable request.
