# Supplementary material for: 210Po and 210Pb content in the smoke of Heated Tobacco Products versus Conventional Cigarette smoking
Source: Sci Rep. 2022 Jun 20;12:10314. doi: 10.1038/s41598-022-14200-2 (PMC9207432; doi:10.1038/s41598-022-14200-2)
Supplement: Supplementary file 1 — Supplementary Information. [file 41598_2022_14200_MOESM1_ESM.docx]

**^210^Po and ^210^Pb content in the smoke of Heated Tobacco Products versus Conventional Cigarette smoking**

Aurélie Berthet, PhD^1^, Audrey Butty, MD ^1^, Jérémie Rossier, PhD^2^, Isabelle Jacot Sadowski, MD^1^, Pascal Froidevaux, PhD^2*^

^1^University of Lausanne, Ctr Primary Care & Publ Hlth Unisante, Lausanne, Switzerland

^2^Institute of Radiation Physics, Lausanne University Hospital and University of Lausanne, Lausanne, Switzerland


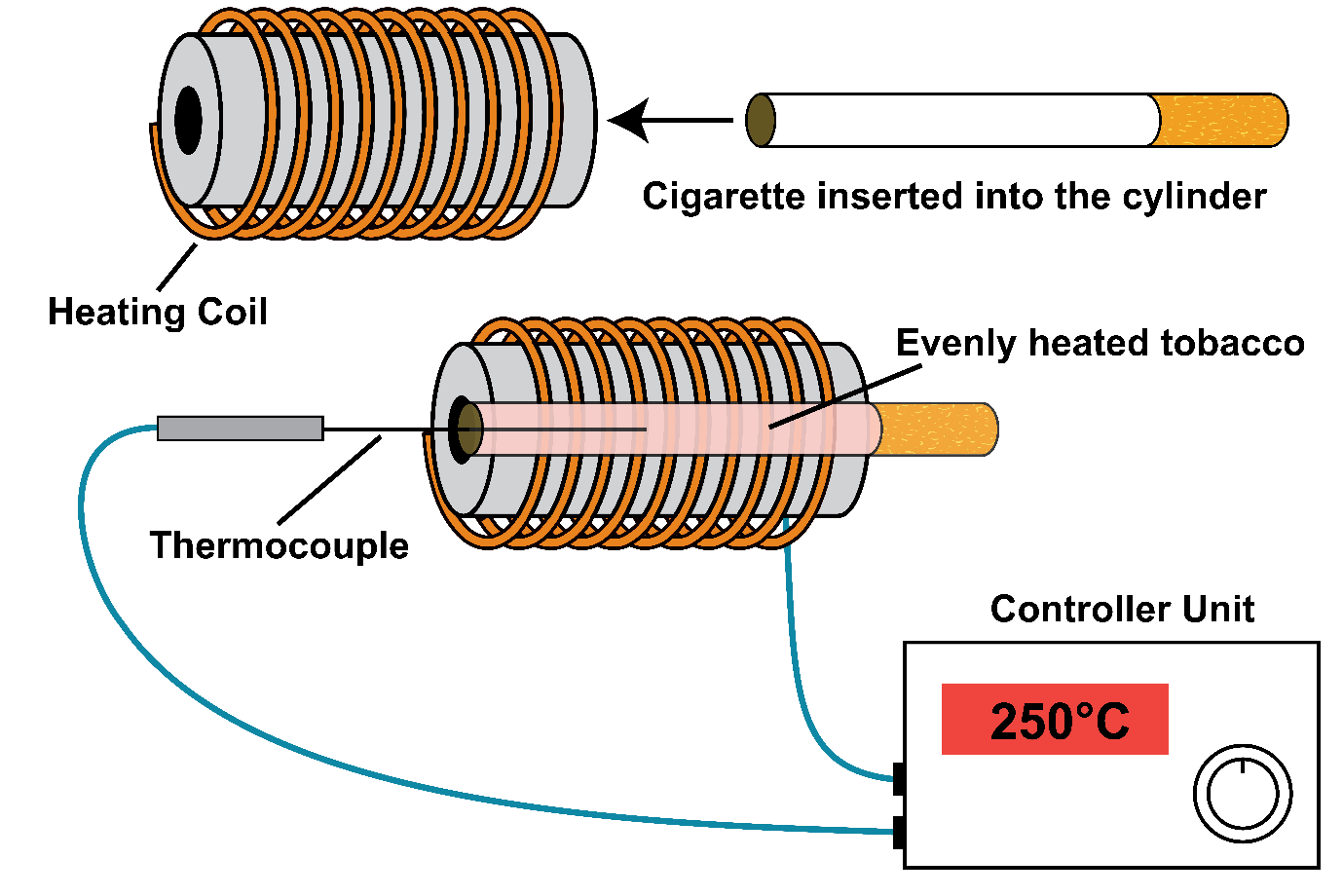


Figure SI1. Scheme of the homemade system for homogenous heating of tobacco. The cigarette is inserted into the copper tube, leaving the filter outside. The copper cylinder is heated using a heating coil at the desired temperature, which is controlled through the thermocouple inserted into the tobacco.
